# Genetic, morphological and growth characterisation of a new *Roseofilum* strain (Oscillatoriales, Cyanobacteria) associated with coral black band disease

**DOI:** 10.7717/peerj.2110

**Published:** 2016-06-09

**Authors:** Patrick Buerger, Carlos Alvarez-Roa, Karen D. Weynberg, Sebastien Baekelandt, Madeleine J.H. van Oppen

**Affiliations:** 1 Australian Institute of Marine Science, Townsville, QLD, Australia; 2 College of Science and Engineering, James Cook University, Townsville, QLD, Australia; 3 AIMS@JCU, James Cook University, Townsville, QLD, Australia; 4 Australian Research Council (ARC) Centre of Excellence for Coral Reef Studies, Townsville, QLD, Australia; 5 Catholic University of Louvain, Louvain-la-Neuve, Belgium; 6 School of BioSciences, University of Melbourne, Melbourne, VIC, Australia

**Keywords:** Cultivation, Coral disease, BBD, Cyanobacteria isolation, Microbial mat, *Roseofillum reptotaenium*, *Pseudoscillatoria coralii*, Great Barrier Reef, Australia

## Abstract

Black band disease (BBD) is a common disease of reef-building corals with a worldwide distribution that causes tissue loss at a rate of up to 3 cm/day. Critical for a mechanistic understanding of the disease’s aetiology is the cultivation of its proposed pathogen, filamentous cyanobacteria (genus *Roseofilum*). Here, we optimise existing protocols for the isolation and cultivation of *Roseofilum* cyanobacteria using a new strain from the central Great Barrier Reef. We demonstrate that the isolation of this bacterium *via* inoculation onto agar plates was highly effective with a low percentage agar of 0.6% and that growth monitoring was most sensitive with fluorescence measurements of chlorophyll-a (440/685 nm). Cell growth curves in liquid and solid media were generated for the first time for this cyanobacterium and showed best growth rates for the previously untested L1-medium (growth rate *k* = 0.214 biomass/day; doubling time *t*_gen_ = 4.67 days). Our results suggest that the trace metals contained in L1-medium maximise biomass increase over time for this cyanobacterium. Since the newly isolated *Roseofilum* strain is genetically closest to *Pseudoscillatoria coralii*, but in terms of pigmentation and cell size closer to *Roseofilum* *reptotaenium*, we formally merge the two species into a single taxon by providing an emended species description, *Roseofilum reptotaenium* (Rasoulouniriana) Casamatta emend. Following this optimized protocol is recommended for fast isolation and cultivation of *Roseofilum* cyanobacteria, for growth curve generation in strain comparisons and for maximisation of biomass in genetic studies.

## Introduction

Coral diseases contribute to coral mortality and to the decline of reefs worldwide (Frias-Lopez et al., 2003; Sutherland, Porter & Torres, 2004; Willis, Page & Dinsdale, 2004; Page & Willis, 2006; Aeby et al., 2015; Bruckner, 2015). Of the over 20 prevalent coral diseases that are known (Sheridan et al., 2013), black band disease (BBD) was one of the first to be described (Antonius, 1973). The BBD microbial community affects a wide range of reef-building coral species and forms a dark mat on the coral surface (Sutherland, Porter & Torres, 2004; Bruckner, 2015), which progresses up to 3 cm per day, and kills the underlying tissue (Rützler, Santavy & Antonius, 1983).

A variety of different bacteria have been identified in the BBD mat, including various species of cyanobacteria, sulphate-reducing *Desulfovibrio* bacteria, *Cytophaga*, *Alphaproteobacteria* and a range of other heterotrophic microbes (Cooney et al., 2002; Sato, Willis & Bourne, 2010; Miller & Richardson, 2011). DNA sequence analysis of the 16S rRNA gene indicates that cyanobacteria of the proposed genus *Roseofilum* (Casamatta et al., 2012), closely related to the genus *Oscillatoria*, are found in the disease mat all over the world (Cooney et al., 2002; Frias-Lopez et al., 2003; Sussman, Bourne & Willis, 2006; Gantar, Sekar & Richardson, 2009; Rasoulouniriana et al., 2009; Sato, Willis & Bourne, 2010; Glas et al., 2010; Miller & Richardson, 2011; Casamatta et al., 2012; Aeby et al., 2015; Arotsker et al., 2015; Meyer et al., 2015). In a non-axenic culture, *R*. *reptotaenium*, is able to cause a progressing BBD lesion in healthy corals a few days after infection (Casamatta et al., 2012). This species has therefore been suggested to play major roles in the aetiology and virulence of the disease (Richardson et al., 2014). However an infection was only possible in the presence of sulfate-reducing bacteria as necessary secondary pathogens (Brownell & Richardson, 2014).

One common approach to study BBD aetiology is to experiment with the dominant BBD cyanobacterium in isolation. To this end, a variety of culture conditions have been applied successfully for the isolation and cultivation of the respective cyanobacteria with ASNIII as the most commonly used culturing medium (Sussman, Bourne & Willis, 2006; Glas et al., 2010; Staniıć et al., 2011; Casamatta et al., 2012; Aeby et al., 2015). However, basic information of an optimised cultivation protocol and BBD cyanobacteria species characterisations were not provided in previous studies, such as growth curves under various conditions, replication times and how to maximise biomass. To fill these gaps, we present an optimized protocol for the cultivation of a *Roseofilum* cyanobacterium associated with BBD that is also applicable to other cyanobacteria present in the disease mat.

## Methods

### Sample collection

Black band diseased coral colonies (*Pavona* sp.) were collected in 3 m seawater depth at Orpheus Island (S 18-34.609/E 146-29.793) in June 2013 (GBRMPA permit G14/36788.1), transported to the Australian institute of Marine Science and maintained in outdoor aquarium systems at 27 °C with shaded, natural sunlight and flow through seawater supply. The isolation of the BBD associated cyanobacteria started during the days immediately following collection.

### Isolation of cyanobacteria

The motile, BBD associated cyanobacteria of the clade *Roseofilum* (Casamatta et al., 2012) were target of this study. Since cyanobacteria live in close symbiotic association with other bacteria, the objective was to produce a viable monoclonal (culture from a single filament), but not axenic culture (may contain other close associated or symbiotic bacteria). Approximately 1 cm^2^ of raw BBD mat was homogenised in 50 mL autoclaved, 0. 04 µm filtered (Memcor ultrafiltration; Siemens, Munich, Germany) seawater by pipetting the slurry up and down with a sterile 1 mL plastic transfer pipette and centrifuged at 3,000 g for 3 min to select and clean the BBD cyanobacteria. The supernatant, containing the majority of other mat associated bacteria was discharged and the cyanobacterial pellet resuspended in autoclaved seawater. The cyanobacterial pellet was inoculated onto an agar plate to clean cyanobacterial filaments from other contaminating microbes (0.6% bacteriological agar in autoclaved seawater, Oxoid, LP0011 agar no. 1) and incubated (Innova 4230, New Brunswick Scientific) under sideway unidirectional light (50–80 µE m^−2^ s^−1^ light intensity) for 6 h at 30 °C (Richardson & Kuta, 2003; Glas et al., 2010). Cyanobacteria migrated towards the light through and on top of the agar while scraping off contaminating bacteria attached to their external cell surface. Those cyanobacteria furthest away from the inoculation site were carefully excised (approx. 2 cm^2^) with a sterile scalpel blade in a biosafety cabinet before being transferred to a fresh, solid agar plate. This cleaning step was repeated twice under the previously described incubation conditions. Subsequently, a liquid culture was established by transferring an approx. 2 cm^2^ agar piece containing a high density of cyanobacteria into freshly prepared medium. After genetic identification of the culture (details below), single cyanobacterial filaments were selected for the establishment of a monoculture as follows: (a) from liquid medium under an inverted microscope and with a micro-pipette; (b) from agar with a stereo-microscope and sterile scalpel. These cultures were grown in liquid L1 medium under a 12 h light and dark cycle at 30 °C with 50–80 µE m^−2^ s^−1^ light intensity (Richardson & Kuta, 2003; Glas et al., 2010). The cyanobacteria were characterised morphologically with an inverted microscope (Leica, DMI 6000 B) and a regular microscope (Zeiss, Axioskop 2 mot plus, 100× oil immersion objective with micrometer reference), monitored visually *via* a stereo microscope (M3Z; Wild Heerbrugg, Heerbrugg, Switzerland) and subcultured if required.

### Genetic identification of cyanobacteria culture

To verify isolation success of the target species, cleaned cyanobacterial filaments were grown in a liquid culture and their DNA extracted from approx. 50 mg of tissue with a Mo-Bio Power Plant Pro Kit (cat. 13400-50) according to the manufacturers recommendations. The V1–V9 region of the 16S rRNA marker gene was amplified with the primers 27f and 1492r (Lane, 1991) in a polymerase chain reaction (MyTaq polymerase Fermentas, cycles: 95 °C for 3 min, 95 °C for 1 min, 55 °C for 1 min, 72 °C for 1 min with a final extension of 72 °C for 5 min). Amplicons were cloned with a TOPO TA Cloning Kit for (pCR 4-TOPO Vector, K4575-02; Thermo Fisher Scientific) and 30 clones were sent for Sanger sequencing with the 27f primer (Macrogen, Seoul, South Korea). Read quality and base calling control was conducted with the software CodonCode Aligner v4.1.1 (CodonCode Corporation, USA). Cyanobacterium isolation success was verified by determination of the closest match for the sequences with a megablast search against the “nr” database at the National Center for Biotechnology Information (www.ncbi.nlm.nih.gov). Sequence variations and close association with other cyanobacteria of the clade *Roseofilum* were visualised in a maximum likelihood tree, generated in MEGA5 (Tamura et al., 2011) with a kimura 2-parameter model and 1,000 bootstrap replications.

### Absorption spectra of photosynthetic pigments

Approximately 10 mg of exponentially growing cyanobacteria filaments were taken from a liquid culture and centrifuged at 10,000 g for 5 min at 4 °C to pellet cells (Eppendorf Centrifuge 5430R). The pellet was subsequently resuspended in 1 mL phosphate buffer (0.1 M) and disrupted in freeze and thaw cycles. Cell debris was pelleted at 10,000 g for 10 min at 4 °C and the supernatant (filtered 0.45 µm) analysed for phycobiliprotein absorbance spectra on a spectrophotometer (Biotek, Synergy H4, 300–700 nm with increments of 1) (Casamatta et al., 2012).

### Solid media preparation and comparison

Cyanobacterial growth on solid medium was compared among three agar concentrations (final agar concentration: agar [%] 0.6; 1; 1.5). In brief, 250 mL of fresh seawater was mixed with bacteriological agar (Oxoid, LP0011 agar no. 1), autoclaved in a 500 mL Schott bottle, cooled to approx. 40 °C and enriched with L1-medium (National Center for Marine Algae and Microbiota, East Boothbay, ME, USA), Table S1. Each petri dish was filled with approx. 15 mL of L1 agar and stored inverted at 30 °C. Cyanobacterial growth on agar was estimated per cm^2^ by averaging filament counts along six radial, equally spaced line transects (originating in the centre of the agar plate, drawn onto the plate towards the wall of the petri dish) and extrapolating the numbers to the overall petri dish area.

### Growth measurement optimisation for liquid media

The growth of cyanobacteria in liquid media was assessed *via* time series measurements in 24-well plates (2 mL in each well) using three approaches: (1) optical density (OD) at 750 nm as a pigment independent measurement (Cirés et al., 2011); (2) fluorescence measurements at 440/685 nm (Held, 2011); and (3) percent coverage of bottom of well.

Optical density and fluorescence readings were averaged from an area scan with 25 measurements per well (Synergy H4; BioTek, Winooski, VT, USA). Cyanobacteria at different growth stages were pelleted at 5,000 g for 5 min, dried overnight at 60 °C and weighed (AD-4 Autobalance; Perkin-Elmer, Waltham, MA, USA) to establish the correlation of “biomass—OD 750” and “biomass—fluorescence.”

Percent coverage values were calculated from images taken of the well bottom with an inverted microscope at standardised settings (DMI 6000 B, 5× magnification objective; Leica, Wetzlar, Germany). The pixel count of cyanobacterial filaments was averaged from five images per well and expressed as percent coverage. Although cyanobacterial filaments were growing on the well bottom and in suspension, the coverage of the well bottom was taken as a proxy for the overall growth.

### Liquid media preparation and comparison

Cyanobacterial growth was compared among four different media for culture optimisation: ASNIII, L1, F/2, and IMK (detailed recipes in Table S1). The growth medium ASNIII was prepared in deionised water with complementary vitamin and trace metal solutions (Rippka et al., 1979). Growth medium L1 (1,000× concentrate, National Center for Marine Algae and Microbiota, Bigelow Laboratory for Ocean Sciences, East Boothbay, Maine 04544 USA), F/2 (50× concentrate; Sigma Aldrich Australia, Castle Hill, NSW, Australia) and IMK (premade powder; Wako Chemicals, Richmond, VA, USA) were prepared in autoclaved seawater according to the manufacturers recommendations. All growth media were used in a final 1× dilution and filter-sterilised before use with a cellulose acetate/cellulose nitrate mixed esters membrane (pore size 0.2 µm, cat. no. 430758; Corning, Corning, NY, USA) with high protein affinity to remove any possible contaminants.

Cultures for media comparison were generated by splitting 20 mL of an exponential growing cyanobacterium culture (growing homogeneous and in suspension) into four equal parts and pelleting the filaments at 3,000 g for 3 min (Allegra X-15R; Beckman Coulter, Brea, CA, USA). The supernatant was discarded and each pellet was resuspended in 10 mL of freshly prepared growth medium: ASNIII; L1; F/2; IMK, respectively. The fresh cyanobacterium stocks were distributed randomly into two 24-well plates (1.5 mL per well, wells per medium = 6) and incubated at 30 °C in a 12 h light cycle at 50–80 µE m^−2^ s^−1^ (PAR) with shaking at 30 rpm. Cyanobacterial growth curves were assessed by conversion of fluorescence measurements over time (440/685 nm) into dry weight and by calculating growth rates *k* = log(10)*X*_*i*_ − *X*_0_∕log(10)2∗*t* (where *X*_*i*_ − *X*_0_ is the biomass difference calculated from the end and start of the exponential phase) as well as doubling times *t*_gen_ = 1∕*k* for exponential phases (Neidhardt, Ingraham & Schaechter, 1990).

### Statistics

Differences in growth curves of cyanobacteria on agar and in liquid media were statistically analysed by comparing regression slopes from log phases with a one-way analysis of variance (ANOVA) and a Tukey post-hoc comparison, all assumptions met (Tables S1 and S5).

## Results and Discussion

### Genetic and morphological characterisation of the isolated cyanobacterium

In this study, we optimised the isolation of the main BBD associated *Roseofilum* cyanobacterium *via* phototaxis on agar, and provide a cultivation method which results in healthy, fast growing and viable filaments (this will not provide an axenic culture, Supplemental Information Note 1).

We successfully isolated the target cyanobacterium, because the 16S rRNA gene sequences of our cyanobacterial culture showed a 99%–100% identity (0–4 nucleotide differences within 346 bp) to the publicly available BBD-associated *Roseofilum* reference sequences. Cyanobacteria of the proposed genus *Roseofilum* are known to be the most abundant cyanobacterial species in terms of biomass within the BBD community (Rasoulouniriana et al., 2009; Sato, Willis & Bourne, 2010; Miller & Richardson, 2011; Casamatta et al., 2012). However, the exact species identification was not as straight forward as the genus classification.

The dominant BBD-associated cyanobacteria were originally classified as *Pseudoscillatoria coralii* Rasoulouniriana et al., 2009. This genus and taxon was argued to be invalid due to incorrect orthography (no Latin description, no type indication) and re-established instead as *Roseofilum reptotaenium* Casamatta et al., 2012 (International Code of Botanical Nomenclature (ICBN) and International Code of Nomenclature of Prokaryotes (ICNP)). Although *P. coralii* and *R. reptotaenium* share >97% of their 16S rRNA gene sequence, they were not considered the same species. Both taxa were maintained in Casamatta et al. (2012) as separate species in the genus *Roseofilum* because of differences in trichome dimensions and associated pigments (Table 1). However, a new name for *P. coralii* has not been established, and a detailed taxonomic revision of *P. coralii* within the genus *Roseofilum* has yet to be undertaken (Casamatta et al., 2012).

**Table 1:** Comparison of cyanobacteria previously isolated from black band disease, *Roseofilum* clade. Note that *Roseofilum reptotaenium* strain AO1 is genetically most similar to strain BgP10_4s from the Red Sea, but morphologically closer to the Caribbean strains. “Host genus” refers to the diseased coral from which the strain was isolated.

*Roseofilum* strain	Trichome/cell width and length (µm)		Cell tip shape	Dominant pigmentation (nm)	Host genus, location	Colour of culture	Clumping & motility	Source
*R. reptotaenium* 100-1	2.5–4.0	3.0–3.9	Round, tapered	Phycoerythrin 548, 565, 620	*Siderastrea*, Caribbean	Dark red/brown	Yes	Casamatta et al. (2012)
*R*. *reptotaenium* 101-1	3.2–3.6	3.4–4.0	Round, tapered	Phycoerythrin 548, 565, 620	*Diploria*, Caribbean	Dark red/brown	Yes	Casamatta et al. (2012)
BDA 82.01	4.0–4.2	4.0–4.5	Round, tapered	Phycoerythrin 548, 565, 620	*Montastrea*, Bermuda	Dark brown/black	Yes	Rützler, Santavy & Antonius (1983)
*R. reptotaenium* AO1	3.6–4.0	3.8–4.3	Round, tapered	Phycoerythrin 548, 568, 620	*Pavona*, GBR	Dark brown/black	Yes	This study
BBD cyanob. isolate	4.0–4.2	Na	Round, tapered	Na	*Montipora,* GBR	Na	Na	Glas et al. (2010)
BgP10_4S (former *P. coralii*)	5.0–6.0	Na	Round, tapered	Phycocyanin 336, 436, 666	*Favia,* Red Sea	Dark green	Yes	Rasoulouniriana et al. (2009)

**Notes.**

Na data not available

**Figure 1:**
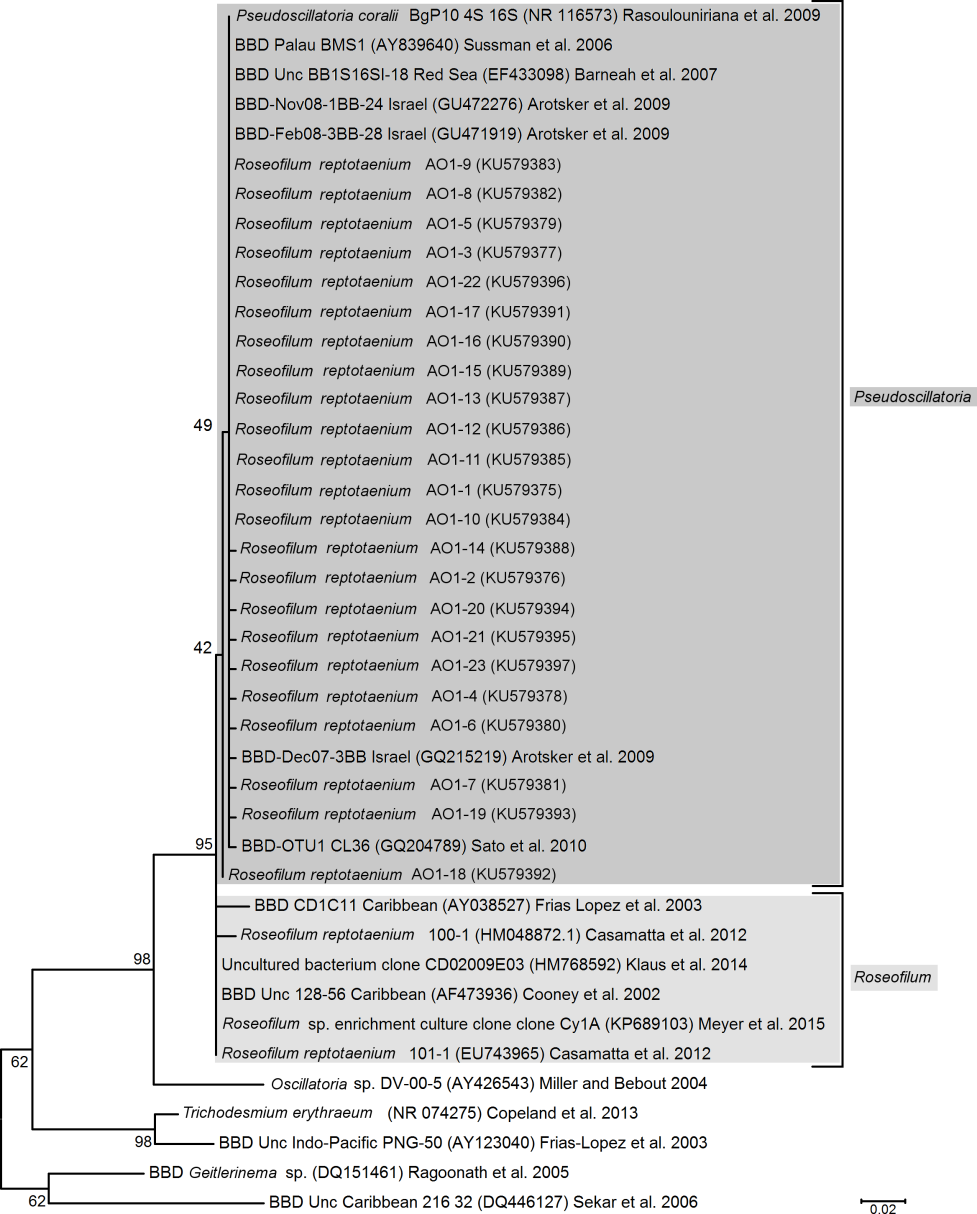
Phylogenetic tree of black band disease cyanobacterial partial 16S rRNA gene sequences based on maximum likelihood analysis. Numbers next to branches indicate percentages of replicated trees in which associated sequences grouped together (bootstrap, *n* = 1,000). Reference cyanobacterium sequences were selected based on close blast matches (cultured cyanobacteria in top 20 blast hits) and previous studies of cultured cyanobacteria. All aligned sequences were trimmed to the shortest reference sequence (346 bp). The scale indicates evolutionary distance, calculated using Kimura 2-parameter in MEGA5.

**Figure 2:**
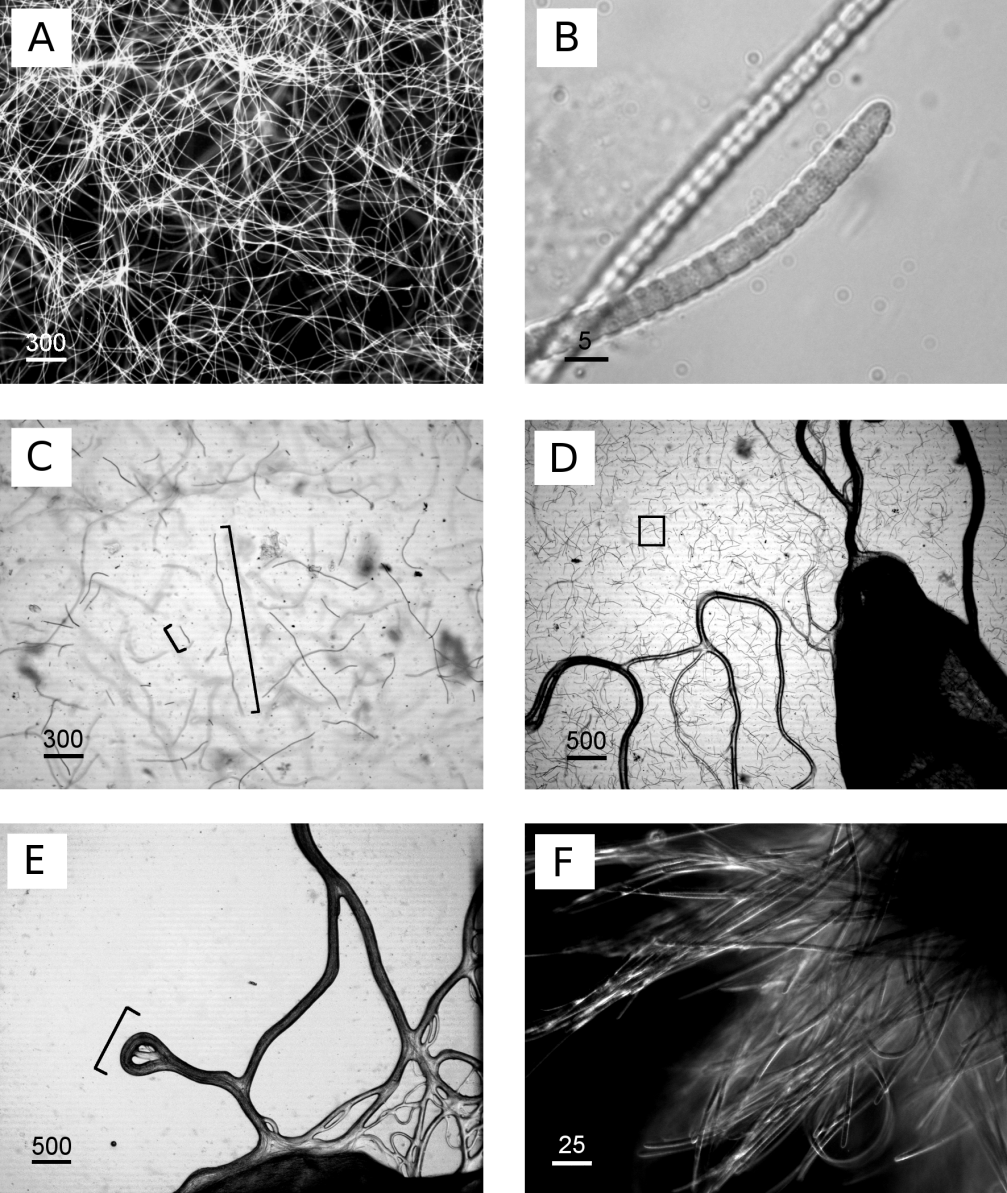
Images of cyanobacterium filament morphologies on agar and liquid cultures. (A) Homogeneous, exponential cyanobacterial growth in L1 medium, filament length up to 1,200 µm, (B) detailed image of isolated cyanobacteria, (C) agar 0.6%, cyanobacteria grew within and not on top of agar, image taken during transition of exponential phase (longer cyanobacteria) to collapse of the culture (shorter cyanobacteria, approx. 100 µm), (D) agar 1%, cyanobacterial grew on top (dark lines) and within agar (square), (E) agar concentration 1.5%, cyanobacterial grew only on top of agar plates in tracks (bracket) in close proximity to the inoculation site without penetrating into the agar, (F) clumps/aggregates of cyanobacteria formed in larger volumes e.g., 250 mL flasks. Scale bars in µm.

**Figure 3:**
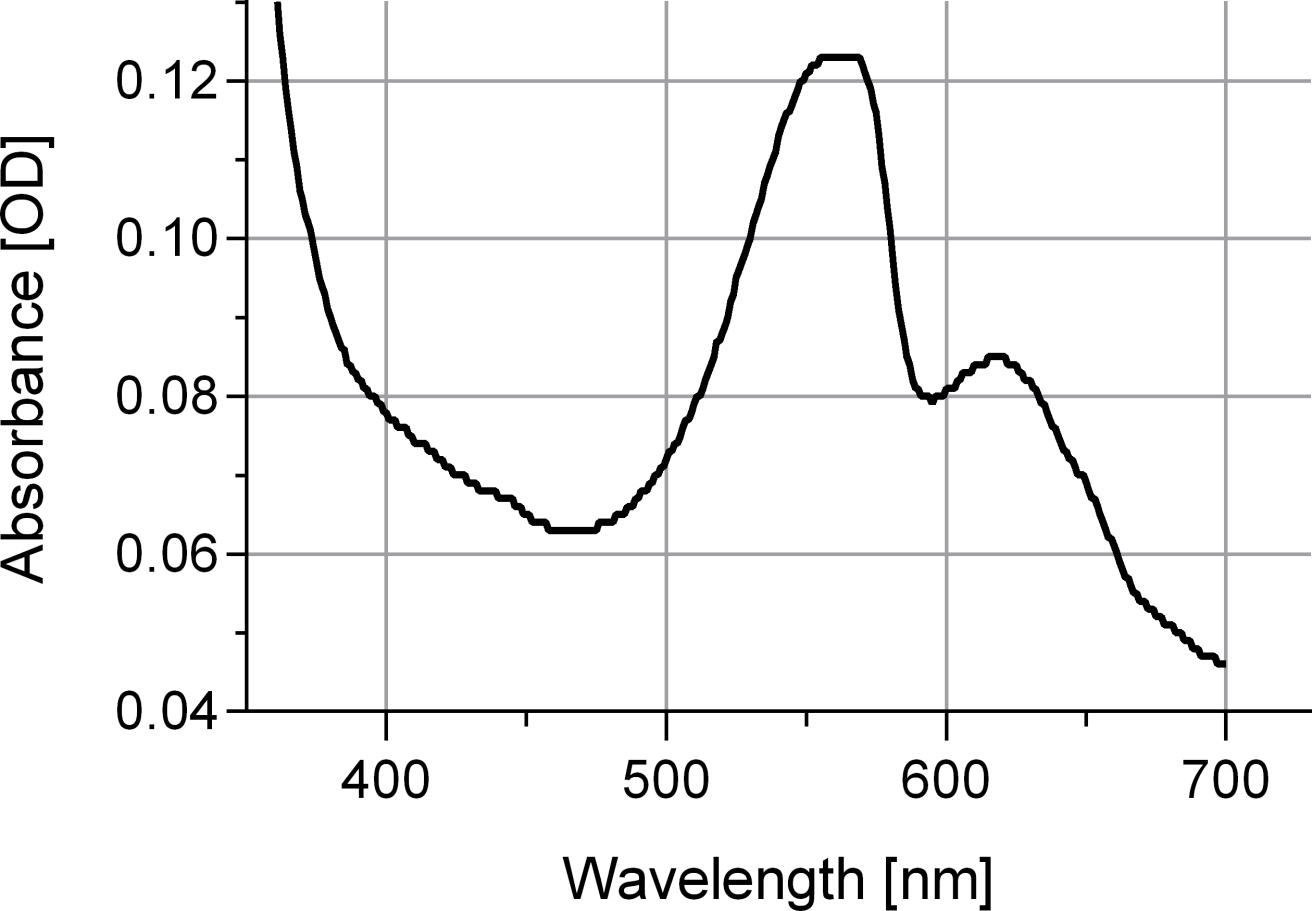
Absorbance spectrum for associated phycobiliproteins. Note representative peaks for phycoerythrin (548 and 565 nm) and phycocyanin (620 nm). Cyanobacteria were grown in L1 medium.

The newly isolated cyanobacterium of the present study showed characteristics of both *P*. *coralii* and *R*. *reptotaenium*. In terms of genetics, the partial 16S rRNA gene sequences of *P*. *coralii* and the newly isolated cyanobacterium were 99–100% identical and clustered separately from *R. reptotaenium* within a phylogenetic tree, albeit with low bootstrap support (bootstrap value = 42, Fig. 1). In terms of morphology, the unbranched trichomes with rounded and tapered cell tips were up to 4 µm in width (Figs. 2A and 2B), i.e., smaller than *P*. *coralii*, but slightly larger than *R*. *reptotaenium* (Table 1). Associated pigmentation was most similar to *R. reptotaenium* due to light absorbance peaks at 620, 548 and 565 nm, indicating the presence of phycocyanin and phycoerythrin respectively (predominantly phycoerythrin due to ratio 565:620 (0.123/0.85) = 1.45; Casamatta et al., 2012) (Fig. 3, Fig. S1; Table 1).

Our results show, that the used characteristics (16S rRNA gene, morphology and pigmentation) that were used to distinguish the BBD-associated *Roseofilum* species do not reliably separate *P. coralii*, *R. reptotaenium* and the cyanobacterium of the present study. The phenotype of strain BgP10_4S is clearly an exception compared to the other reported *Roseofilum* species (Table 1). However, geographical separation, environmental or culture-based pressures may have led to the observed distinct phenotypic diversity among BBD associated *Roseofilum* strains (Dvořák et al., 2015). A multiple DNA marker analysis is required to validate taxonomic affinities among the *Roseofilum* strains (Wu, Jospin & Eisen, 2013) and show if strain BgP10_4S is a true, distinct taxon from *R. reptotaenium* (i.e., *P. coralii* with phycocyanin as the main pigment, lacking phycoerythrin pigments, and exhibiting large cell dimensions of 5–6 µm).

Nevertheless, due to the overlapping characteristics of the current strains of *P. coralii*, *R. reptotaenium* and the cyanobacterium of the present study, the most parsimonious solution is the integration of the species into a single taxon, as already practised in Richardson, Miller & Blackwelder (2015). We therefore provide an emended diagnosis for *R*. *reptotaenium* below and unite the two taxa according to the principle of priority (i.e., the final, valid and earliest taxon description, article 38 and 41 of the International Code of Nomenclature for algae, fungi, and plants ICN), into *Roseofilum reptotaenium* (Rasoulouniriana) ex Casamatta, while *Pseudoscillatoria coralii nom*. *inval*. Rasoulouniriana becomes its synonym. As a consequence, the newly isolated cyanobacterium of the present study has been classified as *Roseofilum reptotaenium* strain AO1 (Australia, Orpheus Island, strain isolate1, Genbank: KU579375–KU579397, collected at Orpheus Island, Australia, S 18-34.609/E 146-29.793 in June 2013, 3 m seawater depth, from black band disease on Pavona *sp*. coral) (Oren, 2011a; Oren, 2011b) and deposited as an epitype (CS-1145) to the Australian National Algae Culture Collection ANACC, Hobart, Tasmania, Australia, www.csiro.au/ANACC. The holotype is deposited at the Brigham Young University Herbarium of Non-Vascular Cryptogams BRY C-53584 (Casamatta et al., 2012).

### Emended description of *Roseofilum reptotaenium*

**Table:** 

Roseofilum reptotaenium (Rasoulouniriana) ex Casamatta, emend
Synonym: *Pseudoscillatoriacoralii nom*. *inval.* Rasoulouniriana et al., 2009

Gram-negative, motile cyanobacterium growing epizoic on corals in black, microbial mats that move over the coral surface and kill the underlying tissue (associated with coral black band disease). In culture, filaments appear can appear dark-green to blackish-brown and reach up to 1 mm in length. Unbranched trichomes with thin sheath, no heterocysts, tapered cells tips, cells of of 3.0–4.5 µm length. High levels of phenotypic plasticity with variants in terms of cell width and pigmentation (Table 1) ranging from 2.5 to 4.0 µm cell width and predominant pigment phycoerythrin in the Caribbean; 3.6–4.0 µm cell width on the Great Barrier Reef; to 5.0–6.0 µm cell width and pigment phycocyanin in the Red Sea. 16S rRNA gene sequence may show minor nucleotide variations depended on the sample location (Fig. 1). Optimal growth conditions from 25 °C to 30 °C, pH 7 and 8, salinity 5–5.5% (*w*∕*v*). For further details access full formal description of the genus *Roseofilum* and the species *R*. *reptotaenium* in Casamatta et al. (2012) Phycologia 51:489–499, and Rasoulouniriana et al. (2009) Dis Aquat Organ 87:91–96.

### Solid media comparison

**Figure 4:**
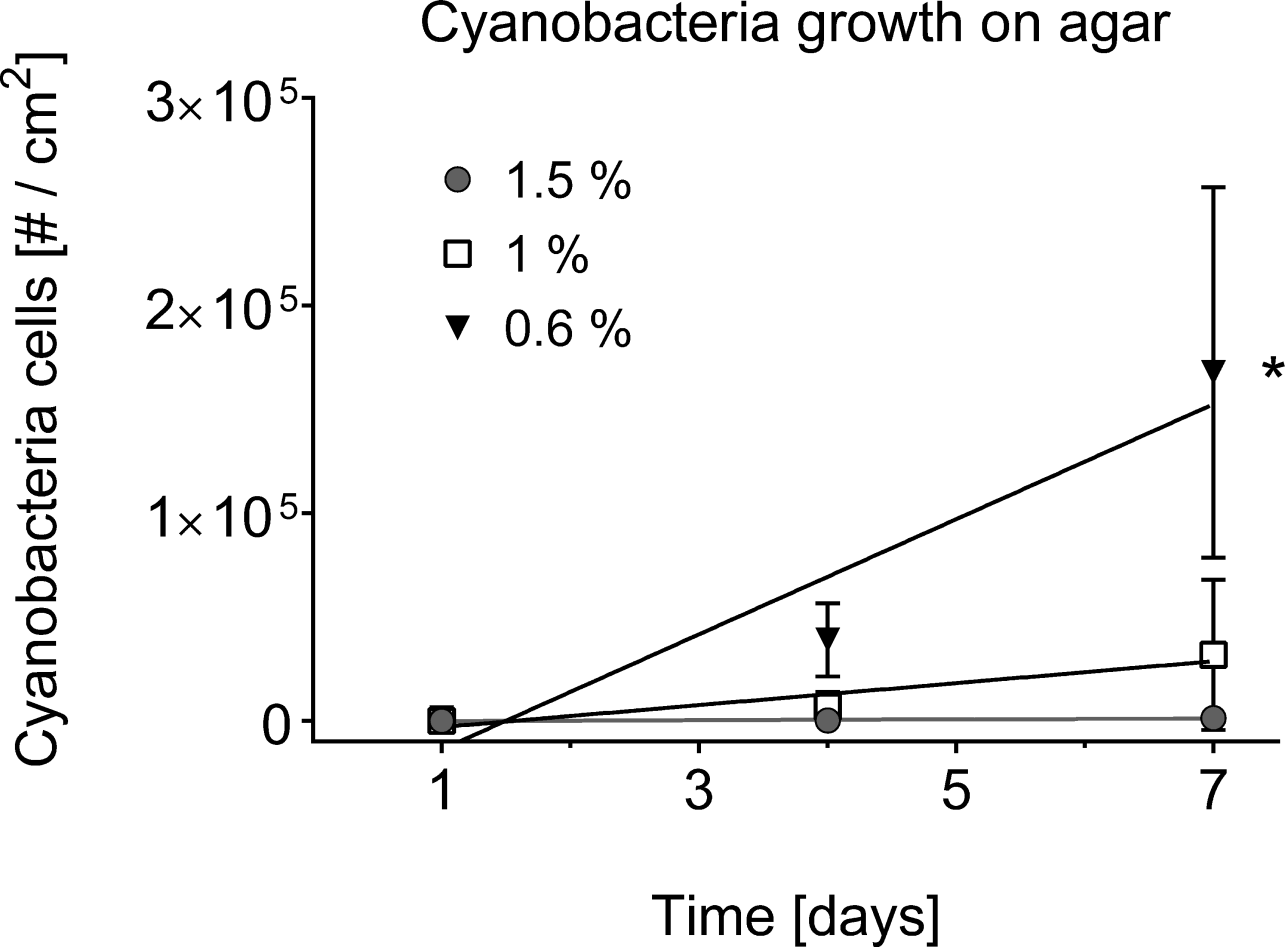
Cyanobacterial growth on various agar concentrations. Cell counts are displayed over time in days. A square centimetre of exponentially growing cyanobacteria in agar was inoculated on each of the plates (*n* = 3 agar plates for every agar concentration) and bacterial cell counts (#) monitored over time. The use of 0.6% agar resulted in a significantly higher cyanobacterial growth rate (indicated by asterisk, 0.6% vs. 1.0%, *p* = 0.0332, at significance level of 0.05) compared to the other agar concentrations of 1% and 1.5%.

The separation of cyanobacteria from the microbial mat was the most time-efficient with motility *via* phototaxis on an agar surface towards a unidirectional light source (Vaara, Vaara & Niemela, 1979). Previous studies that isolated BBD cyanobacteria from the microbial mat did not indicate used agar concentrations (Sussman, Bourne & Willis, 2006; Glas et al., 2010; Stanić et al., 2011; Casamatta et al., 2012; Aeby et al., 2015), but reported e.g., motility over the agar surface with up to 5 cm per day (Sussman, Bourne & Willis, 2006), or almost no motility at all (Glas et al., 2010). The use of a 0.6% soft agar in our study resulted in faster motility (up to 8 cm in 6 h) compared to higher percentage agars (1.5% with almost no gliding at all) (ANOVA Table S3, 0.6% vs. 1.0%, *p* = 0.0325; 0.6% vs. 1.5%, *p* = 0.0186). Cyanobacteria on 0.6% agar spread within the entire agar plate with three times as many filaments after 7 days compared to the next successful treatment of 1% (Figs. 2C–2E and 4). There was no spread or cell replication of cyanobacteria on 1.5% agar plates. A higher percentage and more sticky agar (1.5%) might be more effective to scrape off contaminants from motile cyanobacteria than a lower percentage agar (0.6%) (Rippka, Waterbury & Staier, 1981). However, due to the reduced growth and reduced motility of cyanobacteria on the tested higher percentage agars, we recommend using a lower percentage agar with at least three repetitive steps to clean up filaments.

Two additional cyanobacteria have been isolated with a low percentage agar of 0.6% and deposited into the sequence reference database Genbank the National Center for Biotechnology Information (www.ncbi.nlm.nih.gov). Based on top 5 blast hits (97–99% identity), cyanobacteria species 1 (KU720412) was close related to *Leptolyngbya* sp. (KJ206339.1), *Oscillatoria limnetica* (AF410934.1) and *Phormidium* sp. (JF837333.1), while the second cyanobacteria species (KU720413) was closest to *Limnothrix* sp. (DQ889938.1). These two species were only able to move through a 0.6% agar and could be potentially missed during the isolation process if a higher percentage agar was used.

### Growth measurement optimisation

**Figure 5:**
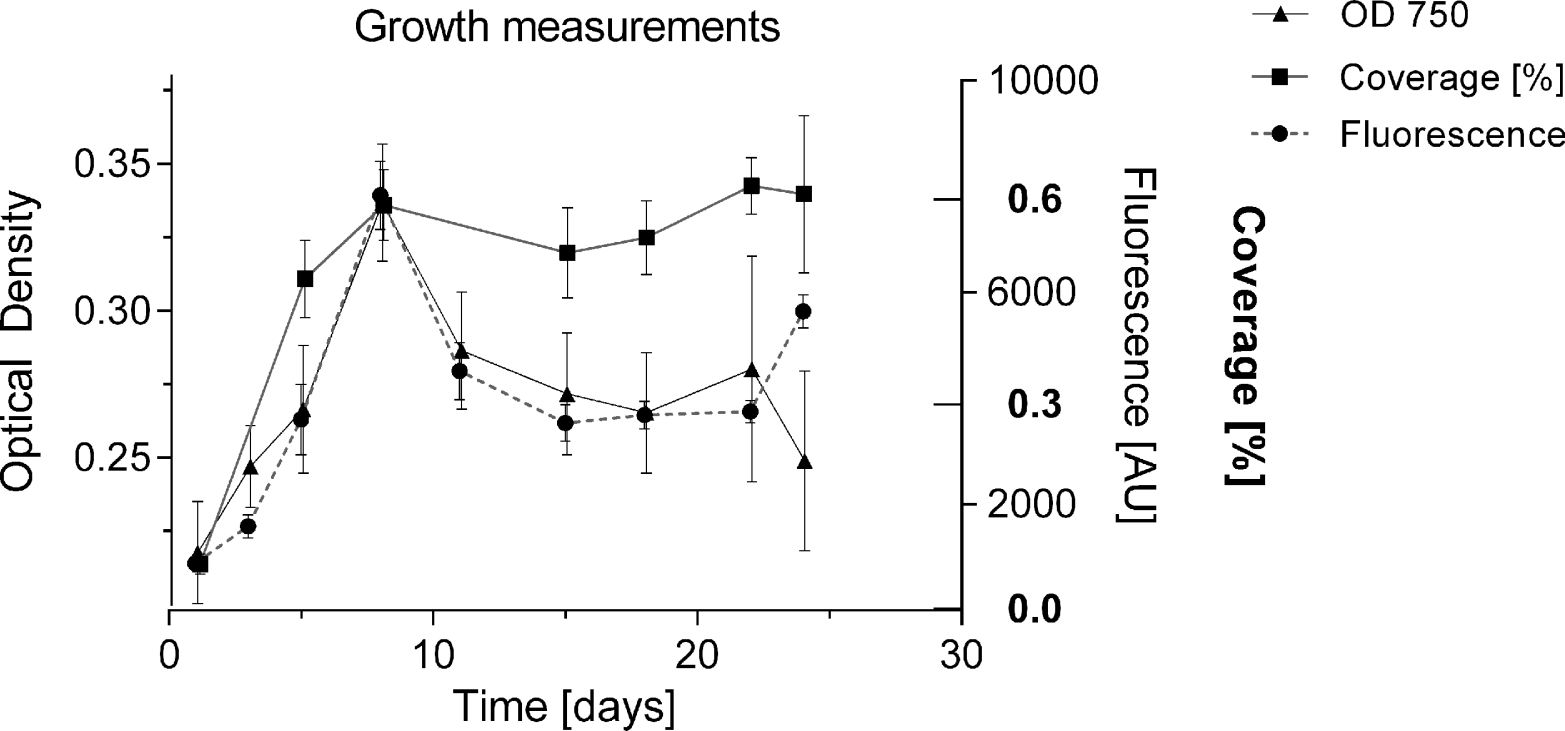
Comparison of methods for measuring growth. Cyanobacterial cell number was monitored for 24 days with different methods (% surface coverage of well bottom; fluorescence of chlorophyll-a 440/685 nm; optical density 750 nm). *Y*-axes of the respective measurements were adjusted to compare methods by setting start and maximum values within the same range.

**Figure 6:**
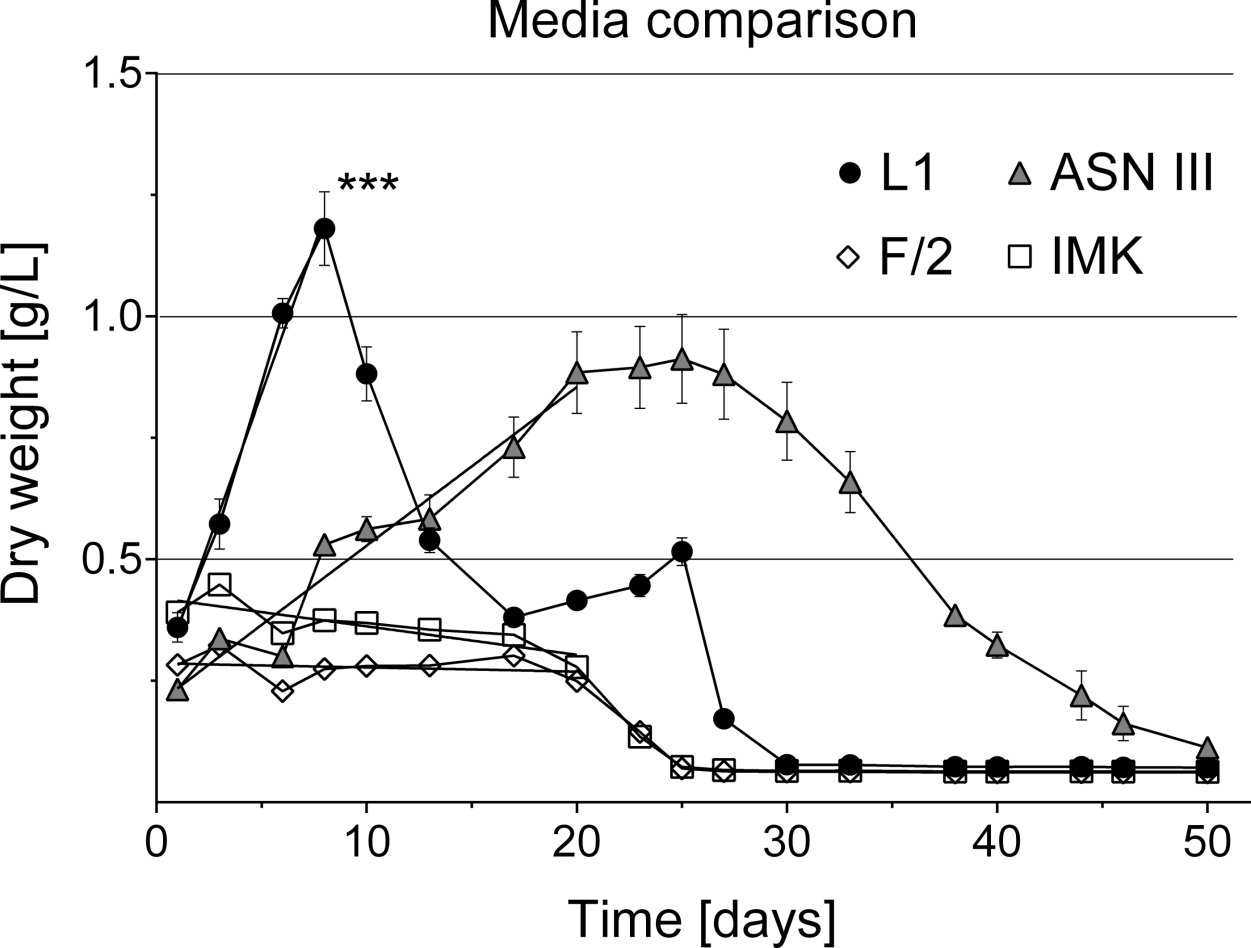
Cyanobacterial growth curves in different liquid culture media. Cyanobacterial growth was tested in four different media types (L1, ASNIII, F/2 and IMK). Cyanobacteria cultures in L1 medium grew exponentially, reached significantly higher cell densities (indicated by asterisk, *p* = 0.0001 at significance level of 0.05) and collapsed earlier without additional nutrient supply than in the other media types (*n* = 6 for each media type, volume 1.5 ml).

To date, growth of BBD associated cyanobacteria has only been qualitatively assessed *via* visual inspection of cultures (Sussman, Bourne & Willis, 2006; Glas et al., 2010), or by biomass measurements following four weeks after inoculation (Gantar, Sekar & Richardson, 2009). The formation of clumps and aggregates (Richardson et al., 2014), with no homogeneous cell distribution makes it difficult to estimate cell numbers and replication in culture. In the present study, the undisturbed cyanobacterium culture in liquid medium showed homogeneous growth with no clumping behaviour in smaller volumes of up to 2 mL per well (Fig. 2A), and could be monitored for cell replication *via* various methods: optical density [OD 750], fluorescence of chlorophyll a (440/685 nm) and well coverage measurements (%) (Fig. 5). All measurements indicated exponential growth from day 1 to day 8. Coverage (%) showed a long stationary phase (approx. duration 16 days) with no decline in cyanobacterium abundance, whereas fluorescence and optical density measurements indicated a decline of the culture (after day 8). Coverage (%) was not considered further, due to unreliable measurements and the inefficiency of this labour-intensive approach. Fluorescence was used for subsequent measurements as it provided a more sensitive detection capability, measuring a second growth peak after 25 days and offered more consistency across measurements (Fig. 6). The linear relationship between biomass (dry weight in g/L) and fluorescence (Fig. S1) (equation: *y* = 0.00011*x* + 0.04863; *r*^2^ = 0.794) allowed the conversion of measured fluorescence values into biomass for the calculation of growth rates (*k*) and doubling times (*t*_gen_) for subsequent experiments and comparison with other strains.

Clumping behaviour of filaments was observed in only after the cultures had been disturbed (e.g., shaking during transportation or growth measurements). However, clumped filaments were capable of homogenising overnight once returned to the constant incubator environment. In addition, previously reported ring formations and continuous clumping behaviour (Richardson et al., 2014) were observed, but only in larger volumes of >250 mL (Fig. 2F).

### Liquid media comparison

Various media types have been compared for the cultivation of cyanobacteria of the *Roseofilum* clade (Glas et al., 2010; Sussman, Bourne & Willis, 2006) with most of the reports of successful growth using ASNIII medium (Richardson & Kuta, 2003; Sussman, Bourne & Willis, 2006; Glas et al., 2010; Casamatta et al., 2012). In the present study, cyanobacterial filaments in ASNIII had a growth rate of *k* = 0.078 biomass/day and time to double dry weight *t*_*gen*_ = 12.85 days, with a survival time of 50 days (Fig. 6). Although ASNIII enabled cyanobacteria to survive longer compared to the other tested media types, we observed reduced motility and short filaments in a similar range as observed for the unsuccessful media types F/2 and IMK (approximately 100–300 µm). Since cyanobacterium species are known for filament length variation and shorter filaments during nutrient depletion (Gibson & Smith, 1982; Smith & Gilbert, 1995; Kruskopf & Du Plessis, 2006), we were particularly interested in a media type that enhanced filament length and increased biomass. Improved growth results were achieved with the media type L1, which, to the best of our knowledge, has not previously been tested to grow BBD cyanobacteria. Cyanobacteria in L1 medium appeared as longer filaments of up ∼1,200 µm in length (Fig. 2C), survived ∼25 days without additional nutrient supply (Fig. 6) and started to grow exponentially within the first day to 8 days after inoculation (growth rate *k* = 0.214 biomass/day; time to double dry weight *t*_gen_ = 4.67 days; ANOVA Tables S4 and S5; slope comparison L1 vs. ASNIII: *p* = 0.0001). Filaments in L1 medium reached twice the amount of biomass after 20 days compared to the ASNIII cultures, if L1 nutrients were re-supplied by inoculation of N, P, trace metals and vitamins with a final × 1 concentration every 3 days (biomass at day 20 for L1 = 1.78 ± 0.2 g, compared to ASNIII = 0.88 ± 0.21 g). Interestingly, the only difference between the chemical components of L1-medium (best growth) and F/2-medium (almost no observed growth) was the presence of selenous acid (H_2_SeO_3_), nickel (II) sulfate hexahydrate (NiSO_4_⋅6H_2_O), sodium orthovanadate (Na_3_VO_4_) and potassium chromate (K_2_CrO_4_) in the former. Due to the differences in growth of cyanobacteria in L1 and F/2 media, it is likely that the presence of one or more of these trace metals is essential for maximising the growth potential of *R. reptotaenium* AO1. Since these trace metals are not present in ASNIII medium (Table S1), it is possible that L1 enhances growth as well for close related Caribbean and Red Sea strains of *R. reptotaenium*, that have so far only been cultivated in ASNIII (Richardson & Kuta, 2003; Sussman, Bourne & Willis, 2006; Glas et al., 2010; Casamatta et al., 2012).

## Conclusion

We present an optimised cultivation protocol for the main BBD *Roseofilum* cyanobacterium and formally unite the taxa *P. coralii* and *R. reptotaenium* in an emended species description into *Roseofilum reptotaenium* (Rasoulouniriana) Casamatta emend. Healthy, fast growing and viable *R. reptotaenium* AO1 cultures were established on a low percentage 0.6% L1 agar (by transferring a dense cyanobacteria agar pellet onto a new plate every 7–10 days) and in L1 liquid medium (250 mL with subculturing every month and reapplication of nutrients to prolong exponential phases and increase biomass). The species isolation with a low percentage agar (0.6%) resulted in faster and easier gliding of cyanobacteria filaments and enabled us to recover two additional cyanobacteria species from BBD samples.

The homogeneous growth of *Roseofilum* filaments in smaller volumes of <5 ml, if undisturbed, allowed the generation of growth curves for the first time for black band disease associated cyanobacteria. Our media comparison showed, that the commonly used growth medium ASNIII did not result in optimal growth conditions while L1 maximised biomass for the tested *Roseofilum* species. Maximising biomass of the cultured cyanobacteria is essential for any downstream genomics, infection experiments, and other culture-based experiments that require replication and a large amount of biomass. Therefore, a standardised culturing method, such as the one provided here, can be critical for ensuring reliable comparisons of morphological, genomic and physiological differences among the isolated black band disease *Roseofilum* cyanobacterial strains.

##  Supplemental Information

10.7717/peerj.2110/supp-1 Supplemental Information 1 Supplementary material

## Additional Information and Declarations

### Field Study Permissions

The following information was supplied relating to field study approvals (i.e., approving body and any reference numbers): Great Barrier Reef Marine Park Authority Permit number: G14/36788.1 to: James Cook University, School of Marine and Tropical Biology, QLD 4811.

### DNA Deposition

The following information was supplied regarding the deposition of DNA sequences: Genbank KU579375–KU579397, KU720412, KU720413.

### Data Availability

The following information was supplied regarding data availability: Figshare: https://figshare.com/s/41a319224ebecfd62eaa.
