# Supplementary material for: Genetic, morphological and growth characterisation of a new Roseofilum strain (Oscillatoriales, Cyanobacteria) associated with coral black band disease
Source: PeerJ. 2016 Jun 9;4:e2110. doi: 10.7717/peerj.2110 (PMC4906641; doi:10.7717/peerj.2110)
Supplement: Supplemental Information 1 [file peerj-04-2110-s001.doc]

**Supplementary information**

Note S1: Our culture was not axenic, since we identified a second taxon in the 16S rRNA sequences of the cyanobacterium cultures (KU720414) which matched an alphaproteobacterium MBIC3865 (AB015896.1, 99% identity, e-value = 0). Cyanobacteria are known to form close associations with epibiotic bacteria, including members of the *Alphaproteobacteria* (Hube et al., 2009; Praveen Kumar et al., 2009). It was therefore not surprising to find this bacterium in our culture. Achieving and maintaining an axenic state of a *Roseofilum* culture may not be possible, because it may rely on the association and presence of symbiotic bacteria (Richardson et al., 2014). Full references in main manuscript.

**Table S1 Media recipes for L1, F/2, ASNIII and IMK.**

**
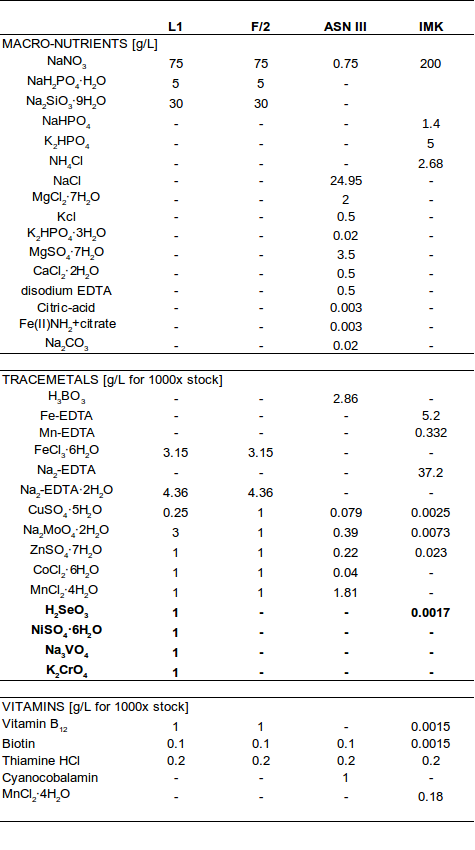
**

**Figure S1 Cell mass absorption spectrum of cyanobacterial culture in liquid medium.** Representative peaks for phycoerythrin (548 and 565 nm) and phycocyanin (620 nm) are marked with arrows in the graph. Measurements and growth in L1 medium.

**
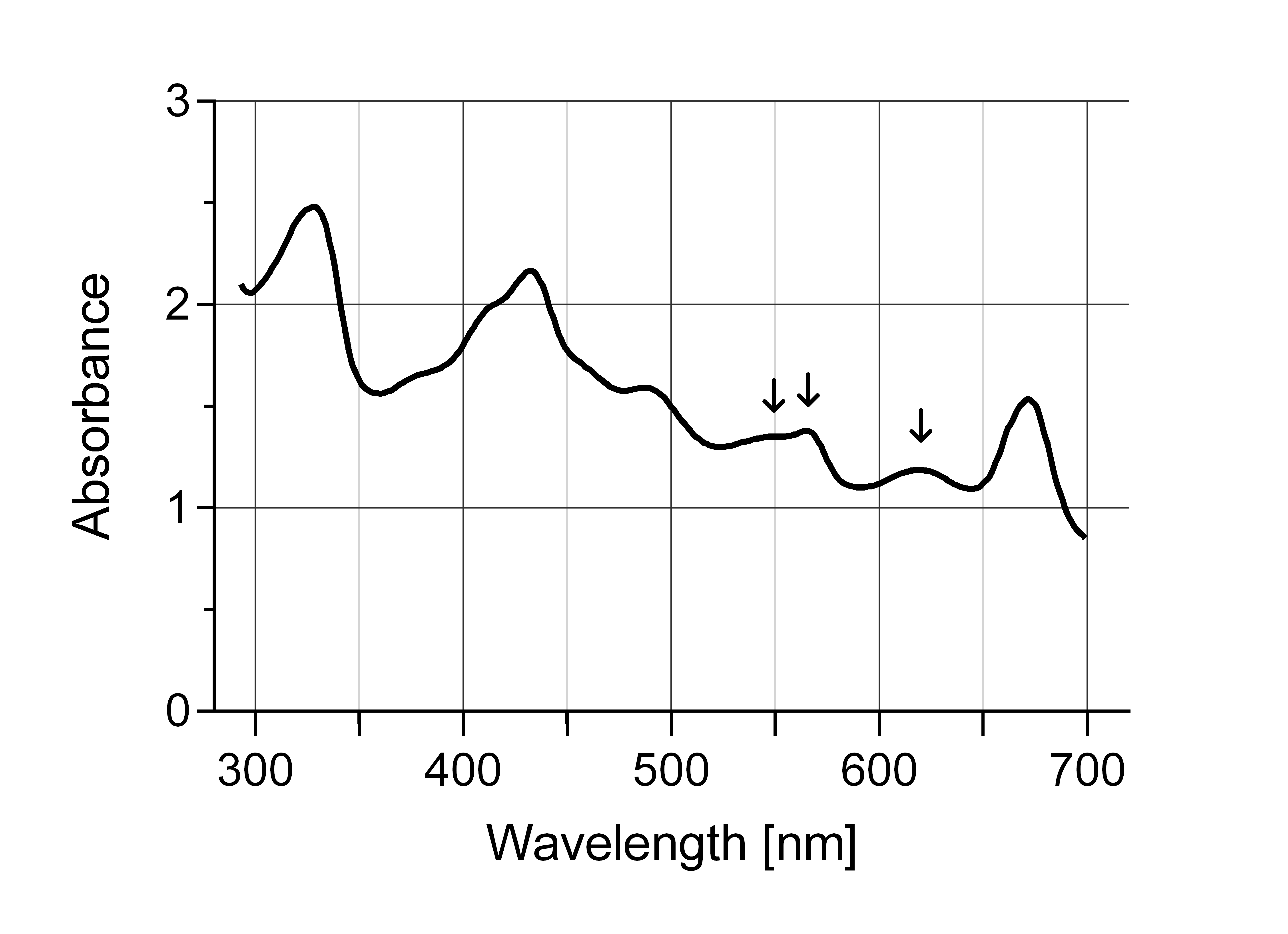
**

**Figure S2 Correlation between fluorescence, OD and biomass (dry weight in g/L).**


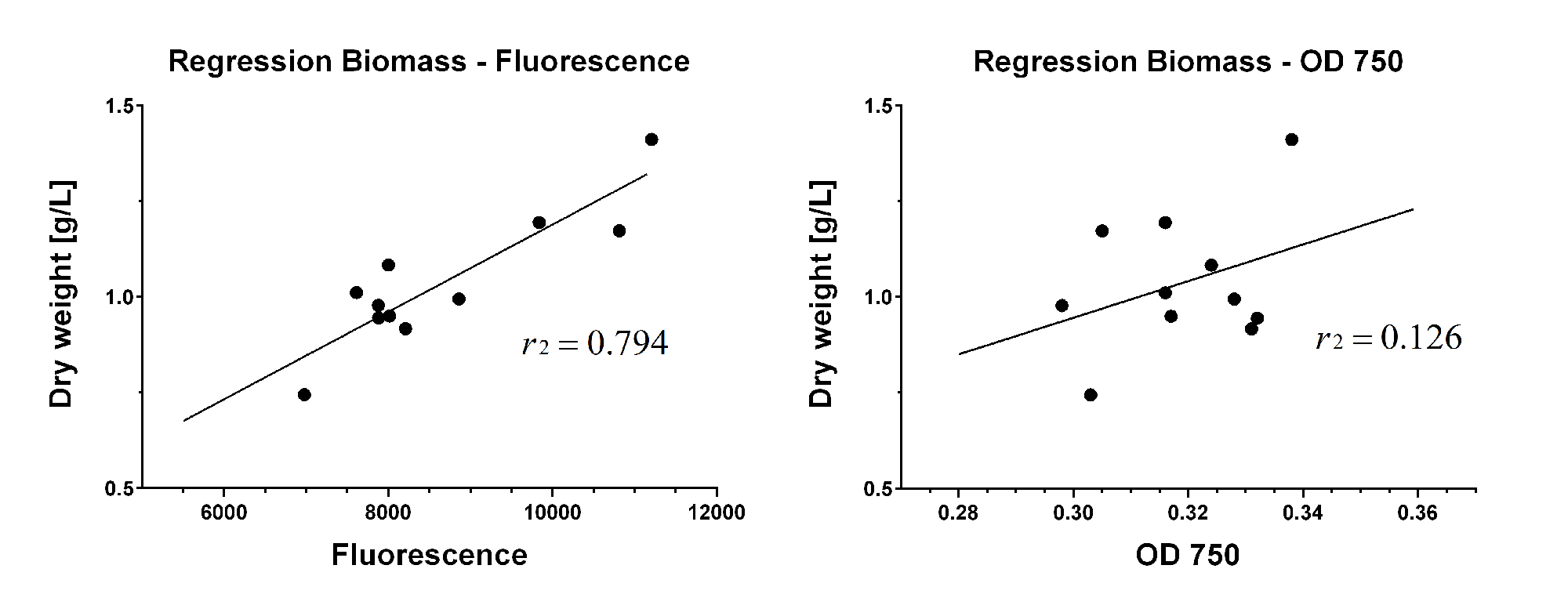


**Table S2 Regression line details of agar comparison.**

| **Agar [%]** | **Slope** | **Different from zero [p-value]** |
| --- | --- | --- |
| 0.6 | 27,732 ± 7,406 | 0.0072 |
| 1 | 5,285 ± 2,763 | 0.0974 |
| 1.5 | 212.8 ± 135 | 0.1589 |

**Table S3 ANOVA details of regression comparison of agar concentrations.** Relevant post-hoc multiple comparisons are written in the results section of the main article.

|  | **SS** | **DF** | **MS** | **F (Dfn, DFd)** | **P value** |
| --- | --- | --- | --- | --- | --- |
| Treatment (between agar conc.) | 8.579 * 10^-8^ | 2 | 4.290 * 10^8^ | F (2, 3) = 20.59 | P = 0.0177 |
| Residual (within agar conc.) | 6.250 * 10^-7^ | 3 | 2.083 * 10^7^ |  |  |
| Total | 9.204 * 10^-8^ | 5 |  |  |  |

**Table S4 Regression line details of liquid medium comparison.**

| **Medium** | **Slope** | **Different from zero [p-value]** |
| --- | --- | --- |
| ASNIII | 0.0326 ± 0.0033 | < 0.001 |
| IMK | -0.0059 ± 0.0017 | 0.0138 |
| L1 | 0.1215 ± 0.0075 | 0.0037 |
| F/2 | -0.0009 ± 0.0017 | 0.6418 |

**Table S5 ANOVA details of regression comparison of liquid medium comparisons.** Relevant post-hoc multiple comparisons are written in the results section of the main article.

|  | **SS** | **DF** | **MS** | **F (Dfn, DFd)** | **P value** |
| --- | --- | --- | --- | --- | --- |
| Treatment (between media) | 0.021 | 3 | 0.0696 | F (3, 4) = 381.7 | P < 0.0001 |
| Residual (within medium) | 7.3 * 10^-5^ | 4 | 0.00015 |  |  |
| Total | 0.021 | 7 |  |  |  |
